# HHV-8 seroprevalence: a global view

**DOI:** 10.1186/2046-4053-3-11

**Published:** 2014-02-12

**Authors:** Eliane Rohner, Natascha Wyss, Sven Trelle, Sam M Mbulaiteye, Matthias Egger, Urban Novak, Marcel Zwahlen, Julia Bohlius

**Affiliations:** 1 Institute of Social and Preventive Medicine (ISPM), University of Bern, Bern, Switzerland; 2 CTU Bern, Department of Clinical Research, University of Bern, Bern, Switzerland; 3 Division of Cancer Epidemiology and Genetics, National Cancer Institute, Bethesda, MD USA; 4 Centre for Infectious Disease Epidemiology & Research, School of Public Health & Family Medicine, University of Cape Town, Cape Town, South Africa; 5 Department of Medical Oncology, University Hospital, Bern, Switzerland

**Keywords:** HHV-8, Kaposi sarcoma, HIV, Seroprevalence, Meta-analysis

## Abstract

**Background:**

Human herpes virus 8 (HHV-8) is the underlying infectious cause of Kaposi sarcoma (KS) and other proliferative diseases; that is, primary effusion lymphoma and multicentric Castleman disease. In regions with high HHV-8 seroprevalence in the general population, KS accounts for a major burden of disease. Outside these endemic regions, HHV-8 prevalence is high in men who have sex with men (MSM) and in migrants from endemic regions. We aim to conduct a systematic literature review and meta-analysis in order 1) to define the global distribution of HHV-8 seroprevalence (primary objective) and 2) to identify risk factors for HHV-8 infection, with a focus on HIV status (secondary objective).

**Methods/design:**

We will include observational studies reporting data on seroprevalence of HHV-8 in children and/or adults from any region in the world. Case reports and case series as well as any studies with fewer than 50 participants will be excluded. We will search MEDLINE, EMBASE, and relevant conference proceedings without language restriction. Two reviewers will independently screen the identified studies and extract data on study characteristics and quality, study population, risk factors, and reported outcomes, using a standardized form. For the primary objective we will pool the data using a fully bayesian approach for meta-analysis, with random effects at the study level. For the secondary objective (association of HIV and HHV-8) we aim to pool odds ratios for the association of HIV and HHV-8 using a fully bayesian approach for meta-analysis, with random effects at the study level. Sub-group analyses and meta-regression analyses will be used to explore sources of heterogeneity, including factors such as geographical region, calendar years of recruitment, age, gender, ethnicity, socioeconomic status, different risk groups for sexually and parenterally transmitted infections (MSM, sex workers, hemophiliacs, intravenous drug users), comorbidities such as organ transplantation and malaria, test(s) used to measure HHV-8 infection, study design, and study quality.

**Discussion:**

Using the proposed systematic review and meta-analysis, we aim to better define the global seroprevalence of HHV-8 and its associated risk factors. This will improve the current understanding of HHV-8 epidemiology, and could suggest measures to prevent HHV-8 infection and to reduce its associated cancer burden.

## Background

Human herpes virus 8 (HHV-8) was first described in 1994 [1], and subsequently identified as the underlying infectious cause of Kaposi sarcoma (KS) [2-5], therefore also called Kaposi sarcoma-associated herpes virus (KSHV). Later, HHV-8 was also linked to other proliferative diseases, including multicentric Castleman disease [6] and primary effusion lymphoma [7]. In 2010, HHV-8 was declared a Group 1 carcinogenic agent by the International Agency for Research on Cancer, highlighting its public health significance [8]. HHV-8 shows a high level of genetic variability, and is classified into five sub-types. Until the advent of the HIV/AIDS epidemic, KS had occurred mainly in older men of Mediterranean and Jewish origin (classic KS) or in equatorial Africa as an endemic form affecting middle-aged men in their fourth or fifth decade. Although HHV-8 is necessary for KS to develop, it is not sufficient, and other cofactors, such as immunosuppression, are needed. Previous studies have shown that immunosuppression is associated with an increased risk of developing KS. In patients with solid organ transplants, the risk of developing KS is increased by a factor of 50 [9], and in untreated HIV-infected individuals, it is increased by a factor of up to 20,000 [10]. Once the HIV/AIDS epidemic began, the incidence of KS increased dramatically and the new, epidemic KS presented with a more aggressive, life-threatening clinical course. From 1996 onwards, combination antiretroviral therapy (cART) was used to reverse HIV-associated immunosuppression, leading to regression of epidemic KS in developed countries [11], and reducing the risk of developing epidemic KS by up to 90% for those receiving cART [12]. Despite this success, the residual risk of developing KS for HIV-infected patients starting cART is still considerable [12]. KS remains a major public health burden in resource-limited countries that have a high prevalence of both HHV-8 and HIV and limited access to cART [13,14].

HHV-8 seroprevalence varies between different geographic regions and sub-populations. For example, in Uganda, where KS is endemic, HHV-8 seroprevalence rates of 50% have been reported for the general population [15,16], whereas, seroprevalence rates of 6% or lower have been reported for the USA [17] and Switzerland [18]. Whitby *et al.* documented regional differences in Italy; a low prevalence was reported for the northern part, whereas a prevalence of 35% was detected in Sicily [19]. This is supported by earlier reports of high incidence rates of classic KS in this region [20]. Ablashi *et al.* also noted wide variation in the detection of HHV-8 antibodies in populations in the USA, Asia, the Caribbean, and Africa [21]. Outside HHV-8 endemic regions, high HHV-8 antibody prevalence has been described in men who have sex with men (MSM) [4,5,18,22] and in migrants from African regions [23]. There is some evidence that in these populations the prevalence of HHV-8 is increasing [24,25]. The association between HHV-8 and HIV seropositivity, especially in heterosexual populations, is a matter of ongoing debate, with some studies showing an association [24,26,27], whereas others do not [15,28,29]. Other risk factors for HHV-8 infection, including lack of circumcision [29], rural residency [30,31], lower socioeconomic status [32], and malaria parasitaemia [33] have been proposed, but remain controversial.

The exact routes of HHV-8 transmission are currently unclear. Both sexual and non-sexual modes have been suggested, but their relative importance may vary with HHV-8 endemicity [34]. In the USA, seroprevalence of HHV-8 is very low in children [35], but, as described above, substantially elevated in certain adult sub-populations such as MSM. In this setting, it is associated with a higher number of sexual partners and a history of previously acquired sexually transmitted diseases (STDs) [4]. By contrast, studies from HHV-8 endemic African countries have revealed a high HHV-8 seroprevalence in infants, indicating horizontal transmission of HHV-8 between parents and children and between siblings in these regions [16,36-38]. The highest levels of HHV-8 shedding are found in saliva [39,40]. Thus, saliva could serve as a medium for HHV-8 transmission, compatible with both sexual and non-sexual pathways.

Two important limitations to the comparability of the HHV-8 seroepidemiological studies conducted to date are the differences in the types of populations studied and the sensitivity and specificity of the serological tests used [3,41]. The populations studied include both selected high-risk groups, such as commercial sex workers, patients attending STD clinics, and miners, as well as members of the general population. With respect to HHV-8 testing, there are various immunofluorescence assays (IFAs) and enzyme-linked immunoassays (EIAs) available, which detect antibodies that are expressed during the latent phase or the lytic cycle of the infection. Although inter-assay correlation is less of an issue in sera with high titers of virus antibodies (for example, sera from patients with KS), results tend to differ in sera from low-risk groups, such as blood donors [41].

To date, one systematic review and meta-analysis has been conducted on the prevalence of HHV-8 and the association of HIV in China. Zhang *et al.* documented a considerable variation in HHV-8 seroprevalence in different geographical sub-regions and ethnic groups, and a threefold increased risk for HHV-8 seropositivity in HIV-infected compared with HIV-uninfected individuals [42]. However, the review was restricted to China, and has methodological limitations; for example, study quality and differences in the characteristics of the HHV-8 tests used were not considered in the analysis.

Our systematic review and meta-analysis will build an important basis for ongoing and future research on HHV-8 and KS. The ultimate goal is to contribute to the development of measures to prevent HHV-8 infection and reduce its associated cancer burden.

### Aims and objectives

We aim to conduct a systematic literature review and meta-analysis to define the global distribution of HHV-8 seroprevalence. We will identify risk factors for HHV-8 infection with a focus on HIV status, and describe changes in HHV-8 seroprevalence over time.

The primary objective is to describe the prevalence of HHV-8 in children and adults stratified by geographical region, age, gender, ethnicity, HIV status, sexual orientation, and calendar year. The secondary objective is to determine the association between HIV and HHV-8 seropositivity in children and adults stratified by geographical region, different risk groups, and calendar year.

### Hypotheses for the analytical part

We will investigate the following three hypotheses: 1) the seroprevalence of HHV-8 will be higher in HIV-infected than in HIV-uninfected individuals; 2) the difference in HHV-8 seroprevalence between populations from HHV-8 endemic and non-endemic regions will be large for HIV-uninfected indviduals but small for HIV-infected individuals; and 3) outside HHV-8 endemic regions, the HHV-8 seroprevalence will have increased over calendar years in high-risk groups such as MSM and HIV-infected individuals, but not in low-risk groups, such as the HIV-uninfected general population and blood donors.

## Methods/design

We will conduct a systematic literature review and meta-analyses using state of the art methods [43]. Reporting will be in accordance with the Preferred Reporting Items for Systematic Reviews and Meta-Analyses (PRISMA) statement [44].

### Study eligibility

#### Primary objective

We will include studies reporting data on the seroprevalence of HHV-8 in populations from any region in the world. Studies measuring HHV-8 DNA only and not HHV-8 antibodies are not eligible. Populations with different risk profiles for HHV-8 infection will be included without restriction. Data for all age groups will be included. We will include cross-sectional, cohort and case–control studies, as well as clinical trials documenting the seroprevalence of HHV-8 with a minimum sample size of 50 analyzed participants in total. Case reports and case series will be excluded. We will primarily seek to include population-based studies, but will also include hospital-based studies. We will include full text publications, but will also assess studies published only as abstracts. Clinical and methodological differences between studies will be assessed in subgroup analyses and meta-regressions (see Statistical analysis section).

#### Secondary objective

The same criteria as detailed above will apply, but we will include only studies that report the prevalence and association of HIV and HHV-8 in HIV-infected and HIV-uninfected individuals sampled from the same source population and tested for HHV-8 antibodies using the same test. Single-arm studies in which HHV-8 seroprevalence has been measured either in HIV-infected or in HIV-uninfected individuals only will be excluded.

### Search strategy for identification of relevant studies

#### Electronic searches

Preliminary electronic literature searches for relevant studies have already been conducted in December 2012. We searched MEDLINE and EMBASE without language restrictions for published reports from 1994 to 2012. An update of the literature search will be conducted in 2014. All literature searches are restricted to studies in humans. The following search strategies are being used.

### MEDLINE search strategy using PubMed

The search terms are: ((((((prevalen* OR inciden* OR epidemiolog* OR seroprevalen* OR sero-prevalen* OR seroepidemiolog* OR sero-epidemiolog* OR seropositiv* OR sero-positiv*)) OR ((((seroepidemiologic studies[MeSH Terms]) OR prevalence[MeSH Terms]) OR incidence[MeSH Terms])))) AND (((((((((((((herpesvirus 8, human[MeSH Terms]) OR “human herpesvirus 8”) OR “hhv-8”) OR “hhv8”) OR “kshv”) OR “kaposi sarcoma associated herpesvirus”) OR “kaposi’s sarcoma associated herpesvirus”) OR “kaposi sarcoma-associated herpesvirus”) OR “kaposi’s sarcoma-associated herpesvirus”) OR “kaposi sarcoma herpesvirus”) OR kaposi virus))))) NOT ((animals[mh] NOT humans[mh])).

### EMBASE search strategy using science direct

The search terms are as follows:

#1.1 “human herpesvirus 8”/exp OR “human herpesvirus 8” OR “hhv-8” OR “hhv8” OR “kshv” OR “kaposi sarcoma associated herpesvirus”/exp OR “kaposi sarcoma associated herpesvirus” OR “kaposi sarcoma-associated herpesvirus”/exp OR “kaposi sarcoma-associated herpesvirus” OR kaposi NEXT/3 herpesvirus OR kaposi NEXT/3 virus AND [embase]/lim

#1.2 “prevalence”/exp OR prevalence OR “seroprevalence”/exp OR seroprevalence OR “incidence”/exp OR incidence OR “seroepidemiology”/exp OR seroepidemiology AND [embase]/lim

#1.3 prevalen* OR inciden* OR epidemiolog* OR sero*epidemiolog* OR sero*prevalen* OR sero*positiv* AND [embase]/lim

#1.4 #1.2 OR #1.3

#1.5 #1.1 AND #1.4

#1.6 “animals”/exp NOT “humans”/exp

#1.7 #1.5 NOT #1.6

#### Searching other sources

We will also contact experts in the field to identify additional and unpublished studies. We will screen reference lists of relevant papers and search the following conferences:

•Annual Meeting of the American Society of Clinical Oncology (ASCO) ( http://www.asco.org/ASCOv2/Meetings);

•International Conference on Malignancies in AIDS and Other Acquired Immunodeficiencies ( http://www.capconcorp.com/meeting/2013/14thICMAOI/index.asp);

•Conference on Retroviruses and Opportunistic Infections (CROI) ( http://www.croi2014.org).

#### Selection of studies

Two reviewers will independently screen the study references identified through the literature search for the eligibility criteria stated previously. If eligibility cannot be assessed from the title and abstract, we will obtain a full version for assessment. Studies that appear to meet the inclusion criteria in the initial screening will be further assessed by scrutinizing the full text reports and publications. If there is insufficient information to judge eligibility, the principal investigator of the study will be contacted for clarification. Any disagreement between the two reviewers will be resolved by discussion. All steps of the literature search will be documented and illustrated in a flow diagram.

### Data extraction

Two reviewers will use a standardized and piloted form to extract data on study characteristics, study population, exposures, risk factors, and reported outcomes. Study characteristics will include the study design and quality, calendar year of sample collection, geographical location, study population, and setting. Data on study population and setting will include country, location of household (rural versus urban), age, gender, ethnicity, socioeconomic status (for example, number of household members, crowding, water source, shared toilet), sexuality (sexual orientation, sexual practices, past STDs, circumcision), HIV status and use of cART, different risk groups for sexually and parenterally transmitted diseases (MSM, sex workers, hemophiliacs, intravenous drug users (IDUs)), other causes of immunosuppression (for example, solid organ transplantation), and other comorbidities such as malaria. We will also document the type of sample tested (serum, plasma, whole blood, other) and the characteristics of the test used to determine HHV-8 seropositivity, including sensitivity, specificity, type of test (IFA, EIA) and antigens used (latent (for example, ORF73/LANA), lytic (for example, ORF65, K8.1, ORF26)). We will extract crude data on prevalence of HHV-8 in different populations with 95% confidence interval (CI), and on factors used for stratifications and adjustments. To evaluate the association of HIV and HHV-8, we will extract the prevalence of HHV-8 in HIV-infected and HIV-uninfected populations and, if available, crude and confounder-adjusted odds ratios (ORs) with 95% CI. Factors used for adjustment will be recorded. Data will be entered into an electronic database to be developed for this project. Data entries of the two reviewers will be compared and revised until consensus is reached. If data are not reported for relevant risk strata, the study authors will be contacted and asked to provide additional information. The effect of missing data will be assessed.

### Quality assessment

In compliance with the National Institute for Health and Clinical Excellence (NICE) [45] and Grading of Recommendations Assessment, Development, and Evaluation (GRADE) [46] guidelines, we will use the criteria listed below to assess risks of bias in observational studies. The effect of these criteria on the overall results will be tested individually; we will not use quality scores because these themselves tend to introduce bias [47]. Because many risk factors for HHV-8 seropositivity have been described, which may act as potential confounders, it is particularly important to assess whether the outcomes of interest have been adjusted or stratified for relevant confounders.

### Quality assessment

Quality assessment will assess the following factors:

1. Study design

a. Study type (cross-sectional study, cohort study, and for the secondary objective, case–control study also).

b. Whether the study design was retrospective or prospective.

2. Research question

a. For the primary objective*:* whether HHV-8 prevalence was the primary outcome of the study.

b. For the secondary objective*:* whether the association between HIV and HHV-8 was the primary outcome of the study.

3. Selection of participants / eligibility criteria

a. Whether the sampling method allowed for a representative sample of the source population

b. Whether inclusion and exclusion criteria were appropriate.

c. Whether participants’ characteristics and exposures with a potential influence on HHV-8 seropositivity were assessed.

e. Whether comparisons are presented between participants and non-participants and whether these were similar. Additionally for the secondary objective:

d. Whether comparability of the HIV-infected and HIV-uninfected group in terms of relevant confounders and effect modifiers was assessed.

f. Whether inclusion and exclusion criteria were the same for HIV-infected and HIV-uninfected people.

4. Measurement of exposures and outcome

a. Whether the test used to assess HHV-8 seropositivity is regarded as valid (the following tests are regarded as valid: IFA, EIA).

b. Whether methods to ascertain participants’ characteristics and exposures were both valid and reliable. Additionally for the secondary objective:

c. Whether the same sample (serum, plasma, whole blood, other) was tested in both the HIV-infected group and the HIV-uninfected group.

d. Whether outcome assessors were blinded to the HIV status of the participants.

e. Whether methods to measure potential confounding factors and effect modifiers were both valid and reliable.

5. Control of confounding and interaction (for the secondary objective only).

a. Whether outcomes were adjusted or stratified for relevant confounders.

6. Appropriateness of analytical methods.

a. Whether the statistical method used was appropriate for the outcome data.

b. Whether ≥ 80% of the people included had their samples analyzed.

c. Whether comparisons between individuals who had their sample analyzed and indviduals who dropped out of the study are presented, and whether they were similar.

### Statistical analysis

For the primary objective (prevalence of HHV-8) we aim to pool data on HHV-8 prevalence using a fully bayesian approach for meta-analysis with random effects at the study level [48]. For every estimate of HHV-8 prevalence in the meta-analysis, the number of HHV-8 positive individuals out of all tested individuals will be assumed to follow a binominal distribution with unknown underlying risk, p. We will model the baseline log odds of HHV-8 seropositivity (that is logit(p) as a normal random variable drawn from a common normal distribution) with the mean equal to the baseline log odds in the population of possible studies, and variance representing the variability between studies. We expect moderate to high statistical heterogeneity between studies. Therefore, we aim at exploring differences in prevalence between studies in sub-group analyses, and if feasible, in random-effects meta-regression. Depending on the degree and nature of heterogeneity identified, we might not present a single overall pooled estimate, but rather provide sub-group estimates.

We will explore the following factors that might explain between-study heterogeneity: geographic location of the study, rural versus urban sites, calendar years of recruitment, age, gender, ethnicity, HIV status, different risk groups for sexually and parenterally transmitted infections (MSM, sex workers, hemophiliacs, IDUs), other reasons for immunosuppression (such as solid organ transplantation), study design and size, study quality, and type of HHV-8 test used. Based on epidemiological and biological reasoning, we will assess a limited number of these factors in random-effects meta-regression. Sub-group specific prevalence estimates are required for these analyses. These might be available both within and between studies. Depending on the available number of studies, we will account for this in the analysis by introducing additional random effects.

We expect that different HHV-8 tests have been used across the identified studies. It is known that these tests perform differently in terms of sensitivity and specificity [49-51]. We will perform meta-regression analysis to explore the relationship between test performance characteristics and prevalence. As a sensitivity analysis, we will also perform standard random-effects logistic regression analysis for all analyses mentioned above. HHV-8 prevalence rates will be presented as geographical maps. Individual study estimates will be presented as proportions with 95% Wilson score CI. For pooled estimates, proportions will be accompanied by 95% credibility interval (CrI) and 95% prediction interval (PI). The PI can be interpreted as the range of likely estimates for future studies, and it also reflects between-study heterogeneity.

For the secondary objective (association of HIV and HHV-8) we aim to pool ORs for the association of HIV and HHV-8 using a fully bayesian approach for meta-analysis with random effects at the study level [48]. As a sensitivity analysis or in cases where a bayesian approach is not feasible, we will perform standard random-effects logistic regression analyses. To address confounding we will pool crude ORs as well as adjusted ORs (if reported). The following factors will be considered as potential confounders and sources of bias at study level: age, gender, ethnicity, sexuality (sexual orientation, sexual practices, past STDs, circumcision), different risk groups for sexually and parenterally transmitted infections (MSM, sex workers, hemophiliacs, IDUs), socioeconomic status (for example, number of household members, crowding, water source, shared toilet), location of household (urban, rural), cART use, other causes of immunosuppression (for example, solid organ transplantation) and comorbidities such as malaria. We will explore sources of heterogeneity between studies with prespecified sub-group analyses and determine differences between sub-groups with meta-regression. The following factors will be considered: geographic location of study, calendar years of recruitment, age, gender, ethnicity, use of cART, sexual orientation, different risk groups for sexually and parenterally transmitted infections (MSM, sex workers, hemophiliacs, IDUs), study design and size, study quality and type of HHV-8 test used. Individual study estimates will be presented as ORs accompanied by standard 95% CI. For pooled estimates, ORs will be accompanied by 95% CrI as well as PI.

Continuous covariates will be modeled using fractional polynomials, with a maximum of two power terms. Selection of power terms will be based on the deviance. All bayesian analyses will be performed using WinBUGS v.1.4.3. For the mean of the random-effects distribution, weakly informative prior distributions will be used. In many situations, the available data make it difficult to estimate the between-study heterogeneity that is captured by the standard deviation, τ [52,53]. Thus, the results for the posterior distribution of τ can be sensitive to the choice of the prior distribution for τ. We therefore will conduct sensitivity analyses with different choices for the prior distributions of τ. The required burn-in will be determined using three chains and subsequent plots of the Brooks-Gelman-Rubin statistics and auto-correlation plots [54]. We plan to use 50,000 iterations as posterior distribution, but the final decision will be based on the size of the Monte Carlo error (< 0.001). All standard random-effects analyses and data management will be performed using Stata v.12.1 (StataCorp, College Station, TX, USA); maps will be realized in ArcGIS v.10.

## Discussion

Since the first description of KSHV (or HHV-8) almost 20 years ago, many studies using different tests have been conducted to measure its seroprevalence in different populations, to identify risk factors for HHV-8 infection, and to investigate possible routes of transmission. Overall, the results of these studies have created a puzzling picture without clear recommendations on the primary prevention of HHV-8 infections.

Therefore, we believe that a systematic review and meta-analysis of the existing studies, taking into account the differences in study design and conduct, is needed. We aim to describe the global distribution of HHV-8 seroprevalence and its associated risk factors. This will provide a valuable basis for ongoing and future research on this topic, and also help to identify research gaps. Until effective and affordable vaccines against HHV-8 become available [55], preventive measures against the spread of this oncogenic virus are needed. The ultimate objective of the project is to contribute to the development of such preventive measures in order to reduce the cancer burden caused by HHV-8 infection.

## Abbreviations

cART: Combination antiretroviral therapy; HHV-8: Human herpesvirus 8; HIV: Human immunodeficiency virus; IDU: Intravenous drug user; KS: Kaposi sarcoma; KSHV: Kaposi sarcoma-associated herpesvirus; MSM: Men who have sex with men; STD: Sexually transmitted disease.

## Competing interests

The authors declare that they have no competing interests.

## Authors’ contributions

JB and ER drafted the protocol with support from UN and SMM. ER, JB, and NW developed the search strategy, the eligibility criteria, and the preliminary data extraction form. ST drafted the statistical analysis section, and MZ and ME gave input on scope of the project, data extraction, and the statistical analyses. All authors read and approved the final version of this protocol.
